# Recent Update of HDAC Inhibitors in Lymphoma

**DOI:** 10.3389/fcell.2020.576391

**Published:** 2020-09-03

**Authors:** I-Chung Chen, Bidyadhar Sethy, Jing-Ping Liou

**Affiliations:** School of Pharmacy, College of Pharmacy, Taipei Medical University, Taipei, Taiwan

**Keywords:** lymphomas, HDAC inhibitors, tumor immunity, chemotherapy regimen, clinical trials

## Abstract

Modulating epigenetic modification has been recognized for over a decade as an effective therapeutic approach to cancer and many studies of histone deacetylase (HDAC), one of the best known epigenetic modulators, have been published. HDAC modulates cell proliferation and angiogenesis and plays an essential role in cell growth. Research shows that up-regulated HDACs are present in many cancer types and synthetic or natural HDAC inhibitors have been used to silence overregulated HDACs. Inhibiting HDACs may cause arrest of cell proliferation, angiogenesis reduction and cell apoptosis. Recent studies indicate that HDAC inhibitors can provide a therapeutic effect in various cancers, such as B-cell lymphoma, leukemia, multiple myeloma and some virus-associated cancers. Some evidence has demonstrated that HDAC inhibitors can increase the expression of immune-related molecules leading to accumulation of CD8 + T cells and causing unresponsive tumor cells to be recognized by the immune system, reducing tumor immunity. This may be a solution for the blockade of PD-1. Here, we review the emerging development of HDAC inhibitors in various cancer treatments and reduction of tumor immunity.

## Introduction

Epigenetic modification plays an important role in regulating gene expression without changing deoxyribonucleic acid (DNA) sequence (Yoo and Jones, 2006). Recently, much evidence has shown that histone function, modulated by various types of reversible modifications, such as methylation and acetylation, is crucial in heritable deliverance and cancer progression. Among these modifications, histone acetylation which is controlled by histone acetyl transferase (HAT) and especially, histone deacetylases (HDAC) are regarded as effective fields of cancer therapy (Jenuwein and Allis, 2001; Li and Seto, 2016; Sanaei and Kavoosi, 2019).

In general, histone acetylation is related to chromatin expression. HATs free chromatin through acetylation of histone lysine tails, producing HDACs which oppose this effect (Berger, 2007). Human HDACs have 18 highly conserved members. Based on their functions and analogies to yeast, HDACs can be divided into two families and four classes, a zinc-dependent family (Class I, Class IIa, Class IIb, and Class IV) and a nicotinamide adenine dinucleotide (NAD)-dependent family (Class III) (Seto and Yoshida, 2014). In addition to histone deacetylation, HDACs have also been found to regulate acetylation of a variety of non-histone proteins (Choudhary et al., 2009). The balance between acetylation and deacetylation is often upset in cancer, and expression of aberrant HDACs may lead to inactivation of tumor suppressing genes. On this basis, many compounds has been identified as HDAC inhibitors (HDACI). These include hydroxamic acids, benzamides, short-chain fatty acids and cyclic peptides, all of which modulate overexpression of HDACs in cancer (Noureen et al., 2010). These HDACIs have marked effects on cancer cells where they induce apoptosis, arrest cell cycles and even modulate the immune system (Hull et al., 2016). Here, we review usage of HDACIs in reduction of lymphomas and tumor immunity.

## Lymphoma

Lymphoma and leukemia are blood cancers, and while they share some common symptoms, they have different origins. Leukemia typically begins in the bone marrow, and lymphoma generally develops in the lymphatic system. The lymphatic system, including marrow, spleen and lymph nodes, are part of the immune system, which helps to protect against infection.

Hodgkin’s (HL) and non-Hodgkin’s lymphoma (NHL) are the two main subtypes of lymphoma. HL, a relatively aggressive lymphoma, is characterized by the presence of very large cells known as Reed-Sternberg (RS) cells, which can be classified into two main types: classic Hodgkin lymphoma (cHL) and nodular lymphocyte-predominant Hodgkin lymphoma (NLPHL) (Table 1). On the other hand, in the view of the Leukemia and Lymphoma Society (LLS), NHL is broadly categorized into two groups: B-cell lymphomas and natural killer (NK)/T-cell lymphomas (Table 2). NHL is nine times more common than HL, and there are more than 60 subtypes of NHL, some “aggressive” (fast-growing) and others “indolent” (slow-growing). This classification determines the treatment options.

**TABLE 1:** Classification of Hodgkin lymphomas (cHL).

Hodgkin Lymphoma (10% of all lymphomas)	Classical Hodgkin lymphomas: 95% of HL
	Nodular sclerosis cHL
	Mixed cellularity cHL
	Lymphocyte depleted cHL
	Lymphocyte-rich cHL
	Nodular lymphocyte-predominant Hodgkin lymphoma: 5% HL

**TABLE 2:** Classification of Non-Hodgkin lymphomas.

**Mature B-cell lymphomas: 85% of NHL**	
Non-Hodgkin Lymphoma (90% of all lymphomas)	
Aggressive	Burkitt/Burkitt-like lymphoma
	Diffuse large B-cell lymphoma (DLBCL)
	Double-hit lymphoma
	Mantle cell lymphoma (MCL)
	Primary mediastinal B-cell lymphoma
Indolent	Chronic lymphocytic leukemia/small lymphocytic lymphoma (CLL/SLL)
	Follicular lymphoma (FL)
	Lymphoblastic leukemia/lymphoma (more commonly derived from T cells)
	Lymphoplasmacytic lymphoma/Waldenstrom macroglobulinemia (WM)
	Marginal zone lymphoma (MZL)
**T cell lymphoma: 15% of NHL**	
Aggressive	Anaplastic large cell lymphoma (ALCL)
	Angioimmunoblastic T-cell lymphoma (AITL)
	Peripheral T-cell lymphoma (PTCL)
	Lymphoblastic leukemia/lymphoma (less commonly derived from B cells)
Indolent	Adult T-cell leukemia/lymphoma (ATLL)
	Cutaneous T-cell lymphoma (CTCL)
	Mycosis fungoides (MF)

The two principle therapies for lymphomas are radiation therapy and chemotherapy, and stem cell transplantation is another choice in some lymphoma types. Currently, increasing research efforts show that HDAC could be a therapeutic target in lymphomas (Table 3), and this has inspired us to try to understand its mechanism and development.

**TABLE 3:** HDAC related clinical trials started after 2017 for lymphoma treatment.

NCT number	Condition	Intervention	Status	Phase	Start date
NCT03934567	FL	Abexinostat	Recruit	II	20190502
NCT03770000	R/R T-cell Lymphoma	Tenalisib + Romidepsin	Recruit	I/II	20181210
NCT03600441	FL	Abexinostat	Active	II	20180726
NCT03873025	R/R DLBCL	CXD101 + Pembrolizumab	Not yet recruit	I/II	20190313
NCT03820596	R/R Extranodal NKTCL	Sintilimab + Chidamide	Recruit	I/II	20190129
NCT03547700	PTCL	Romidepsin + Ixazomib	Recruit	I/II	20180606
NCT03630731	R/R Extranodal NKTCL	Chidamide	Recruit	II	20180815
NCT04233294	R/R HL	Chidamide + Camrelizumab with or w/o Decitabine	Recruit	II	20200118
NCT04038411	R/R NKTCL	PD-1 Antibody, Chidamide, Lenalidomide Etoposide	Recruit	IV	20190630
NCT04040491	newly diagnosed PTCL	PD-1 Antibody, Chidamide, Lenalidomide Etoposide	Recruit	IV	20190131
NCT04231448	Newly Diagnosed Double-Expressor DLBCL	R-CHOP + Tucidinostat	Not Yet recruit	III	20200108
NCT03373019	R/R DLBCL	Chidamide + R-GDP	Recruit	II	20171214
NCT04337606	R/R NHL	Chidamide + Decitabine + Camrelizumab	Recruit	I/II	20200407
NCT03161223	PTCL	Durvalumab Pralatrexate Romidepsin 5-Azacitidine	Recruit	I/II	20170519
NCT02943642	Mycosis Fungoides	A-dmDT390-bisFv(UCHT1) vs. Vorinostat	Not Yet Recruit	II	20161025
NCT03713320	CTCL, MF	Cobomarsen vs. Vorinostat	Active	II	20181019
NCT03936153	R/R DLBCL	abexinostat	Recruit	II	20190503
NCT03939182	DLBCL, MCL	Abexinostat + Ibrutinib	Recruit	I/II	20190506
NCT04024696	NHL	Abexinostat	Recruit	I/II	20190718
NCT03179930	R/R lymphoma	Entinostat + Pembrolizumab	Recruit	II	20170607
NCT03150329	R/R DLBCL, FL, or HL	Vorinostat + Pembrolizumab	Recruit	I	20170512
NCT03117751	acute lymphoblastic lymphoma	CHOP Pegaspargase Erwinase^®^ Cytarabine Mercaptopurine Dasatinib Methotrexate Blinatumomab Ruxolitinib Bortezomib Dexamethasone Doxorubicin Etoposide Clofarabine Vorinostat Idarubicin Nelarabine Thioguanine	Recruit	II/III	20170418

*Retrieved from: http://clinicaltrials.gov/.*

## HDAC and T Cell Lymphoma

### Role of HDACs in T Cell Development

HDACs have been reported to be necessary for t cell development. CD4 lineage integrity is regulated by HDAC1 and HDAC2 members through downregulation of Runx3/CBFβ complexes, which induce CD8 lineage programs in CD4 + t cells (Boucheron et al., 2014; Ellmeier, 2015). An HDAC1 and HDAC2 knockout test of t lymphocytes also has been found to result in cell cycle arrest and reduction of thymocytes. These events will eventually lead to decrease of the peripheral t cells and appearance of CD4 + and CD8 + t cells (Preglej et al., 2020). Another experiment with knockout HDAC1 and HDAC2 in mice showed the HDAC1/2-Sin3A-NuRD complex is disrupted. This may block double-negative (DN) to double-positive (DP) transition, and failure of proliferation. Moreover, insufficiency of HDAC1 or HDAC2 may lead to overacetylation of histones and chromosomal instability, finally causing t cell lymphoma (Dovey et al., 2013). Above all, this research indicates that HDAC1 and HDAC2 are essential for t cell development.

HDAC3 has also been found to be indispensable in steps of t cell progression, including commitment of CD4 and CD8, positive selection, and peripheral t cell maturation (Wang P. et al., 2020). HDAC3 deficiency in DP thymocytes terminates CD4-lineage program and redirects the MHC class II-restricted thymocytes toward the CD8-lineage program, due to the acetylation of histone expressing CD8-lineage genes, such as Runx3 and Patz1 (Philips et al., 2019). In a CD2-icre HDAC3 conditional knockout (HDAC3-cKO) mice, t cell development is blocked at the positive selection step, resulting in fewer CD4 and CD8 T cells, and cannot be rescued by TCR-transgene. The absence of HDAC3 renders RORγt unable to down-regulate although positively selected and fails to upregulate Bcl-2, which may lead to apoptosis (Philips et al., 2016). For t cell maturation, HDAC3 forms complexes with NF-kappaB-activating-protein (NKAP), which is necessary for recent thymic emigrants (RTE) to gain functional competency and transfer into a long-lived naive t cell pool. Lack of HDAC3 may cause CD50 downregulation which leads to t cell immaturation. In peripheral T cells, HDAC3 deficiency creates a defect in TNF licensing after TCR/CD28 stimulation (Hsu et al., 2015). Another distinct subset of t cells, nature killer t (NKT) cells, are also HDAC3 dependent during development. As was previously mentioned, NKAP activated through formation of complexes with HDAC3, also participates in invariant NKT (iNKT) cells lineage. Furthermore, HDAC3-deficient iNKT cells show low expression of nutrient receptors GLUT1, CD71 and CD98, and this results in incremental autophagy (Thapa et al., 2016, 2017).

Class IIa HDACs are also involved in t cell development. HDAC5 is implicated in t-regulatory (treg) cells homeostasis. In an HDAC5^–/–^ mice model, T_reg_ cells show reduced suppressive function, CD4 + t cells convert poorly into treg cells, and increasing acetylation of Foxo1 causes treg cells to experience difficulty in maintenance of the phenotype. CD8 + t cells have found to be less able to produce IFN-γ in HDAC5^–/–^ mice (Xiao et al., 2016). HDAC7, a thymus-specific HDAC, acts as a regulator of t cell apoptosis and endothelial cell functions, is highly expressed in DP thymocytes, and inhibits Nur77 that is involved in apoptosis and negative selection. During TCR activation, HDAC7 is exported from the nucleus, leading to Nur77 expression and mediating TCR-mediated apoptosis (Dequiedt et al., 2003; Martin et al., 2008). HDAC6, a class IIb member of the HDACs, controls the production of immunosuppressive cytokine IL-10, and induction of antigen-presenting cells (APCs) that activate antigen-specific naïve t cells through formation of a complex with STAT3 (Cheng et al., 2014). HDAC10, another class IIb HDAC, mediates the inactivation of Foxp3. Foxp3 + treg cells are known to suppress immune responses, and HDAC10 dysfunction may cause some inflammatory disorders (Dahiya et al., 2020). HDAC11, which is the only member of class IV, negatively regulates the expression of IL-10. Overexpression of IL-10 will induce inflammatory APCs, priming naïve t cells and restoring the responsiveness of tolerant CD4 + t cells. Its adjustment with HDAC6 determines t cell activation (Villagra et al., 2009). HDAC11 acts as a positive controller of Foxp3 + tregs, and lack of HDAC11 will increase Foxp3 and TGF-β expression, which may lead to inflammation. The dynamic interaction between HDAC10 and HDAC11 serves to balance the immune response (Huang et al., 2017).

### Evidence of HDACs in T Cell Lymphomas

Investigating the elaboration of HDACs in t cell lymphoma would assist an understanding of their pathogenesis, prognosis and role as a therapeutic target. Although the precise mechanism underlying this behavior has not been elucidated, it can be investigated through HDACIs.

Gene expression that mediates a balance between HAT and HDAC histone modification is important because it is also marks initiation and progression of cancer cells. HDACs intervene in carcinogenesis through the deacetylation of histone and non-histone proteins (Jenuwein and Allis, 2001). Recent research has shown that HDACs are involved in the expression of numerous oncogenes such as Bcl- xL-, BCL2-, c-Myc, TCRβ and Notch3 (Palermo et al., 2012; Kunami et al., 2014; Loosveld et al., 2014; Stengel et al., 2015). HDACs also participate in cytokine regulation. In the study of cutaneous t-cell lymphoma (CTCL) patients, 30% demonstrated the high affinity of the IL-2 receptor, which can be perturbed by HDACIs (Shao et al., 2002). Furthermore, HDAC1 and HDAC6 were also found to be upregulated in CTCL. This causes excessive secretion of IL-15, which mediates the inflammation that is crucial in CTCL, suggesting this oncogenic loop can be controlled by modulation of HDAC1 and HDAC6 (Mishra et al., 2016).

**FIGURE 1:**
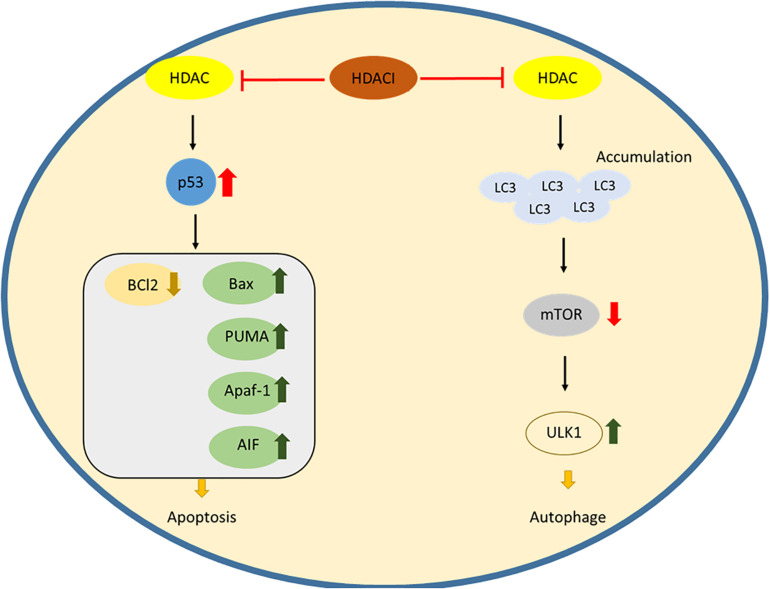
The action of HDAC inhibitors inducing apoptosis and autophagy.

In t cell malignancies, HDACs act as negative controllers of apoptosis, and upregulation of HDACs will silence the pro-apoptotic gene and Bcl2 family expression. During the signaling pathway, HDACs can acetylate the chaperone heat shock protein 90 (HSP90), which stabilizes the client proteins RASGRP1 and c-RAF. These client proteins activate the mitogen-activated pathway, leading to the down regulation of the Bcl2 family (Ding et al., 2017). Upon treatment of peripheral t cell lymphoma with HDACi, chidamide induces cell apoptosis by downregulating Bcl2 and upregulating Caspase3 and Bax protein (Lu et al., 2016). The expression of a tumor suppressor gene was also found to be modulated by HDACs in CTCL cell lines. A combination of an HDACI, romidepsin and a demethylating agent, azacitidine leads to the induction of RHoB, the tumor suppressor gene, and to cell apoptosis (Rozati et al., 2016). The downstream apoptotic pathway regulated by HDACs may serve as a potent therapeutic target for apoptosis induction in a treatment for t cell malignancies (Figure 1).

Another self-devouring process similar to apoptosis is the source of dysfunction in t cell malignancies. Besides deacetylation of lysine residues in the histone, HDACs also have functions in regulation of cytosolic proteins which have a variety of cellular functions, including autophagy. SAHA (vorinostat), a pan-HDAC inhibitor, upregulates the expression of an autophagic factor LC3, inhibiting mTOR, the mammalian target of rapamycin and leading to activation of the autophagic protein kinase ULK1 (Figure 1; Gammoh et al., 2006). HDACs are inseparable by autophagy in cellular survival, and targeting autophagy by inhibition of HDACs could offer an effective treatment for t cell lymphomas.

The main function of HDACIs might be interference with histone and chromatin modification, but acetylation of histone and non-histone proteins may cause DNA damage, expression of suppressing genes in oncogenesis, and either lowering of the apoptotic threshold, or triggering autophagy response. These physiological processes have proved to be indispensable for HDACs in cancer pathogenesis and prognosis, and this makes them a prospective target for t cell malignancies.

### Application of HDACIs in T Cell Lymphoma

#### Vorinostat (SAHA)

**FIGURE 2:**
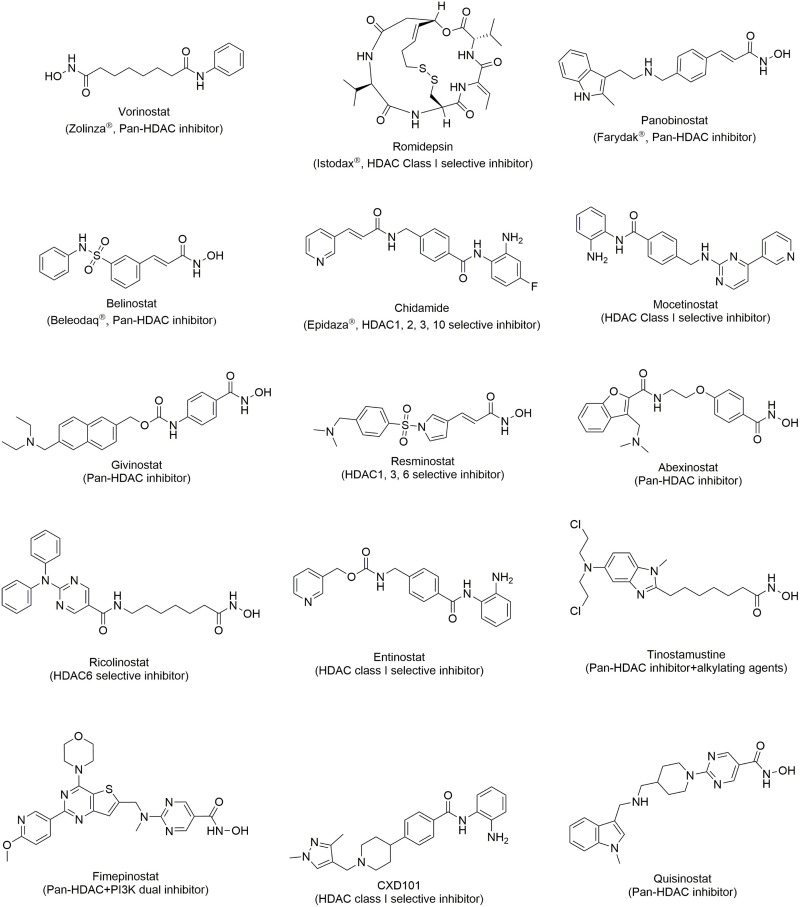
Structure of HDAC inhibitors.

Vorinostat Figure 2, also been known as SAHA, is a hydroxamic acid HDAC inhibitor. It shows inhibitory activity in both class I and class II HDACIs with an IC_50_ less than 86 nM (Molecule of the month, 2006). To date, usage of SAHA has mostly been restricted to the treatment of CTCL. In cellular studies, SAHA shows a surprising anti-proliferative effect on human mantle lymphoma cells, CTCL cells, freshly isolated ATL cells and circulating malignant CTCL cells from patients by upregulating p21, decreasing the phosphorylation level of STAT6, increasing NF-κB in cytoplasm, and arresting the cell cycle (Nishioka et al., 2008; Zhang et al., 2005). In clinical trials, SAHA was tested in patients with refractory and relapsed CTCL with an ORR of 24 or 30% and duration of the response for 4 months or more in two phase II studies. SAHA was approved by FDA in 2006 as a treatment for refractory or relapsed CTCL (Duvic and Vu, 2007; Duvic et al., 2007; Olsen et al., 2007).

In combination therapy, vorinostat combined with azacitidine has been tested. This resulted in 88% event-free and overall survival rates in t cell lymphoma patients (Nieto et al., 2016). In another clinical trial, vorinostat been used in a combination with gemcitabine, busulfan, and melphalan, and demonstrated high efficacy in refractory or poor-risk relapsed t cell lymphomas (Nieto et al., 2015). Vorinostat was also found to increase the effect of rituximab in a phase II study in newly diagnosed or relapsed/refractory NHL patients (Chen et al., 2015). Using a standard cyclophosphamide, hydroxydaunorpubicin, oncovin, and prednisone (CHOP) treatment with newly diagnosed peripheral t cell lymphoma patients in a phase I clinical trial, vorinostat also obtained a good therapeutic effect (Oki et al., 2013b). Combination of vorinostat with PI3K inhibitors or HSP90 inhibitors resulted in cytotoxic antagonism in CTCL cells, and investigation of this could be useful (Wozniak et al., 2010; Hutt et al., 2014). The proteasome inhibitor, bortezomib also causes a synergetic effect inducing apoptosis in CTCL patients (Heider et al., 2009). However, combination therapies do not always give positive results. For example, a study of a combination of lenalidomide, vorinostat, and dexamethasone used to treat patients with relapsed/refractory peripheral t cell lymphoma (PTCL), resulted in median-progression free survival and low overall survival (Hopfinger et al., 2014). Thus, vorinostat is still significant in lymphoma therapy.

#### Romidepsin (FR901228)

Romidepsin Figure 2 is a natural depsipeptide isolated from bacteria. It displays selective inhibition toward class I HDACs but is weak in HDAC IIB (Ueda et al., 1994). The single use of romidepsin, exhibits effectiveness in relapsed/refractory CTCL patients and was approved by FDA in 2009 for the treatment of CTCL (Imam et al., 2013; Reddy, 2016). Romidepsin demonstrates outstanding clinical response in relapsed/refractory PTCL patients and it was also approved in 2011 for PTCL treatment (Barbarotta and Hurley, 2015; Iyer and Foss, 2015; Irlé and Weintraub, 2016; Foss et al., 2017).

In combination therapies, romidepsin has been combined with conventional drugs for hematological malignances, such as methotrexate, vincristine, imatinib, cytarabine, carboplatin, doxorubicin, 4-hydroperoxy-cyclophosphamide, etoposide, 6-mercaptopurine, and SN-38. All of these showed an additive result, indicating that combination therapy with romidepsin is promising. Combining CHOP with romidepsin in newly diagnosed PTCL patients exhibited a surprising therapeutic effect with an overall survival of 71% at the median follow-up of 30 months (Dupuis et al., 2015). Unlike vorinostat, romidepsin shows synergistic effects with lenalidomide in relapsed/refractory lymphomas (Cosenza et al., 2016). Aurora kinase inhibitors are a promising agents for treatment of TCL, and combined with romidepsin their therapeutic effect is highly synergized (Zullo et al., 2014). Clinical trials of this combination are in progress. Combinations of romidepsin with other drugs, including pralatrexate, gemcitabine, and ICE (Wozniak et al., 2010; Sung et al., 2014; Foss et al., 2017) are being studied. Romidepsin seems to be useful in combination therapies, although more investigation is necessary.

#### Belinostat (PXD101)

Belinostat Figure 2 is pan-HDAC inhibitor with a sulfonamide-hydroxamic acid structure. It exhibited nanomolar inhibition against HDACI, II, and IV (Chowdhury et al., 2011). The clinical data from relapsed/refractory PTCL patients shows that belinostat, with high efficacy and low toxicity is an ideal drug for cancer treatment (Campbell and Thomas, 2017). With promising results, belinostat was approved for sale in 2014 for the treatment of relapsed or refractory PTCL (Poole, 2014). Because of its safety, belinostat is a first-line drug for relapsed or refractory PTCL or various drug combination therapies.

For the combination therapies, belinostat has been used with CHOP. In spite of its use as a first-line treatment for relapsed or refractory PTCL, its combination with CHOP delivered a poor prognosis with relapse within 5 years (Johnston et al., 2015). Other usage in combination with bortezomib, volasertib, zidovudine, or carfilzomib, has already been published. Most of these show a potential therapeutic effect, and this provides more alternative options for treatment of lymphoma patients.

#### Panobinostat (LBH-589)

Panobinostat Figure 2 is a cinnamic hydroxamic acid HDAC inhibitor which inhibits HDACI, II and IV and is 10-fold more potent than SAHA (Atadja, 2009). In clinical trials, panobinostat was demonstrated to be effective in patients with advanced CTCL (Duvic et al., 2013). In a clinical trial, panobinostat was acceptably tolerable and led to a modest overall response. However, it failed in the phase II trial due to its low response and short time to progression in refractory CTCL patients (Ellis et al., 2008; McCann and Story, 2013). Panobinostat is now undergoing a clinical trial with PTCL (Tan et al., 2015).

Panobinostat also guided some combination therapies. A combination of panobinostat and bortezomib in PTCL highly synergized the ubiquitination ability in preclinical studies (Samimi et al., 2013). In further clinical studies, a combination of bortezomib displayed promising efficacy, improving the outcome following a single dosage. This Phase III clinical trial for PTCL treatment (Tan et al., 2015) has been completed. In other clinical studies, conspicuously, administration of everolimus and panobinostat to TCL patients decreased serum cytokine levels (Oki et al., 2013a). The severe adverse effects of panbinostat makes its development difficult, but it still offers a new approach for lymphoma therapies. These results in combination with other agents may a sign of a new era of PTCL therapies.

#### Quisinostat

Quisinostat Figure 2 is a broad spectrum HDAC inhibitor, which has strong inhibition activity in HDACs, except for HDAC6, 7, and 9. Its clinical trial for CTCL treatment has been completed in 2016 (NCT01486277). However, the result was failed to be superior comparing to other HDACIs, such as vorinostat or romidepsin while treating with CTCL patients. Furthermore, after dosing quisinostat 5 of 26 patients have grade 3 drug related adverse effect, including, hypertension, lethargy, chills, pyrexia, pruritus and hyperkalemia, even though the safety and tolerability profile is similar to other pan-HDACIs, the overall outcome limited its development (Child et al., 2016). Recently, quisinostat has been combined with bortezomib and dexamethasone for multiple myeloma (NCT01464112), but no any clinical trials in lymphomas.

### Perspective

Recently, HDACIs has been approved for TCL treatment, and most of them belongs to pan-inhibitor, such as vorinostat and belinostat. Indeed, these pan-HDAC inhibitors brought effectiveness in PTCL patients, but not in CTCL patients. Interestingly, romidepsin, a class I HDACs selective inhibitor, showed promising result in CTCL patients. Above all, inhibition of other HDAC subtypes would decrease the efficacy in CTCL, which inspired us targeting class I HDACs might increase the application of HDACI in TCL therapies. Moreover, romidepsin also showed broader availability comparing to other pan-HDACIs in combination with other target therapies or chemotherapies. Thus, further pre-clinical studies are necessary to understand the precise mechanism.

## HDAC and B Cell Lymphoma

### Role of HDACs in B Cell Development

In B cell differentiation, HDAC1 and HDAC2 promote the development in the pre-B cell stage that progresses the cell cycle from G1 to S. Knockout of HDAC1 and HDAC2 leads to cell cycle arrest and expression of p21 and p57, which may cause apoptosis (Yamaguchi et al., 2010). At the terminal stage of B cell development, Blimp-1 restrains c-myc through the aggregation of HDAC1 and HDAC2 (Yu et al., 2000). In HDAC3 knockdown mice, the progenitor B cells cause impaired B cell maturation, and defects in VDJ (varies, diversity, joining) recombination (Stengel et al., 2017). HDACs participate in complex formation in different stages of B cell development. HDACs have been considered to be a component of the STAT5a-LSD1 complex, which demonstrates the possible activation of STAT5a in the early stages of B cell development (Nanou et al., 2017). In mature B cells, Bach2 recruits the HDAC3-NCoR1/NCoR2-Rif1 complex to repress Pdm1 transcription thus blocking the differentiation between B cells and plasma cells (Tanaka et al., 2016).

BCL6, a sequence-specific repressor of transcription, requires formation of complexes with specific HDACs. HDAC3, HDAC4, and HDAC9 have been found to be a corepressor of BCL6 (Lemercier et al., 2002; Pasqualucci et al., 2011; Gil et al., 2016). In the HDAC7 conditional deletion mice experiment, HDAC7 was deemed to control the pro-B cell to pre-B cell transition. In pro-B cells, the transcription factor ME2FC is complexed with HDAC7, which silences the lineage-inappropriate genes, ensuring the correct B cell differentiation (Barneda-Zahonero et al., 2015; Azagra et al., 2016). HDAC6 was also shown to be a controller of PD-L1 in B cells, regulating the immunogenicity (Powers et al., 2014). Selective HDAC6 inhibitors are considered to be a new target for immunotherapy, but the precise mechanism is needed for further investigation.

### Evidence of HDACs in B Cell Lymphoma

In the pathogenesis of lymphoma, HDAC-BCL6 complexes are often aberrant in the transcription step. For instance, the germinal centers (GC) of B cells in CREBBP-regulated/active enhancers are negatively regulated through H3K27 deacetylation by the BCL6-SMRT-HDAC3 complex. In folicullar lymphoma (FL) and diffuse large B cell lymphoma (DLBCL), however, CREBBP mutations lead to unopposed deacetylation by BCL6-SMRT-HDAC3 at an enhancer of B cell signal transduction and expression of immune response genes, which results in lymphomagenesis (Pasqualucci et al., 2011; Jiang et al., 2017). In B-NHL cells, the abnormal expression of HDAC9-BCL6 complex may cause B-lymphoproliferative disorders. Overexpression of HDAC9 contributes to alter pathways involved in growth and survival, as well as modulation of BCL6 activity and p53 tumor suppressor function (Gil et al., 2016). HDAC4 plays a key role in suppressing oncogenes. Dysfunction of HDAC4 disrupts the complex with BCL6 and this may lead to induce uncontrolled proliferation, clonogenic potential, and decreased apoptosis (Lemercier et al., 2002; Sandhu et al., 2012). Targeting these HDACs might therefore have promising effect in B cell lymphoma therapies.

### Application of HDACIs in B Cell Lymphoma

#### Vorinostat (SAHA)

Vorinostat, the first approved HDAC inhibitor, has been used in a phase II clinical trial for relapsed DLBCL therapies. But the overall response rate (ORR) was only 5.6%, which indicates that in single usage, vorinostat is limited (Crump et al., 2008). However, other clinical trials conducted using vorinostat as an FL treatment showed 8-times better ORRs of 47 and 49% (Kirschbaum et al., 2011; Ogura et al., 2014).

In the combination therapies vorinostat, combined with rituximab or R-CHOP in NHL patients, also showed enhanced effects, especially in DLBCL patients with an 81% ORR (Chen et al., 2015; Straus et al., 2015; Persky et al., 2018). A combination of R-ICE and vorinostat for relapsed or refractory NHL patients also had 70% ORR (Budde et al., 2013). Pre-clinical experiments showed that SAHA and topoisomerase inhibitors surprisingly defeated lymphoma cells, and this might be a new aspect for NHL therapies (Seo, 2015).

#### Belinostat (PXD101)

Similar to vorinostat, belinostat behaves poorly in monotherapies. A Phase II clinical trial showed that administration of belinostat to relapsed or refractory aggressive B-NHL patients resulted in only 10.5% ORR (Puvvada et al., 2016). This result terminated the research on monotherapies of belinostat in B cell lymphoma patients. Several combination therapies, however, are still in clinical trials, for example a trial in combination with carfilzomib, a proteasome inhibitor, is still ongoing (NCT02142530).

#### Chidamide

Chidamide Figure 2 is a selective HDAC class I inhibitor, and is now approved only in China (Ning et al., 2012). Its therapeutic effect in relapsed or refractory B-NHL is still being evaluated in clinical trials (NCT03245905 and NCT03410004).

Combination of chidamide with other chemotherapies have also been investigated in clinical trials. Such combinations include R-GDP (NCT03373019), vinorelbine, liposomal doxorubicin, dexamethasone and thalidomide (VDDT) (NCT02733380), dexamethasone and ICE (DICE) (NCT03105596), and R-CHOP (NCT03201471) in relapsed or refractory B-NHL. A clinical trial of R-CHOP combined with chidamide (NCT02753647) in untreated elderly DLBCL patients is progressing (Wang L. et al., 2020).

#### Mocetinostat

Mocetinostat Figure 2 is a selective HDAC I and IV inhibitor, which has been approved by FDA for use in cases of relapsed or refractory CTCL (Boumber et al., 2011). The effect of mocetinostat in a Phase II clinical trial showed low ORR in both DLBCL (18.9%) and FL (11.5%) (Batlevi et al., 2017). These results are similar to those from other HDAC inhibitors, whether selective or not, and show low ORR in B cell lymphoma patients. Therefore, HDACIs may significantly increase the therapeutic effects in combination with other chemotherapies. However, mocetinostat only been used with azacitidine (NCT00543582) in a clinical trial, and further research is necessary.

#### CXD101

CXD101 Figure 2 is a selective class I HDACs inhibitor, which is now undergoing phase I clinical trial to assess its tolerability, safety, pharmacokinetics and pharmacodynamics in advanced malignancies (NCT01977638). It has been hypothesized that selectively inhibiting class I HDACs could reduce the toxicity, which is brought by off target inhibition on class II HDACs. Preliminary result shows that, PXD101 has lower toxicity and higher tolerability than other non-selective inhibitor (Eyre et al., 2019).

Besides, CXD101 also test with the combination of pembrolizumab for R/R DLBCL treatment (NCT03873025), but no any other result has been reported.

#### Ricolinostat (ACY-1215)

Ricolinostat Figure 2 is the only HDAC6 selective inhibitor, which entered clinical trial for NHL therapies (NCT02091063). However, there is no any further development in single agent therapies for NHL.

Surprisingly, ricolinostat has synergizing effect combining with other drug or regimen. Such as ibrutinib (Amengual et al., 2017), carfilzomib (Dasmahapatra et al., 2014), bendamustine (Cosenza et al., 2017), and crizotinib (Liu et al., 2018) in DLBCL or MCL models. These combination therapies showed great potency toward DLBCL and MCL, but its efficacy in human is still under investigation.

#### Fimepinostat (CUDC-907)

Fimepinostat Figure 2, targeting HDAC and PI3K, is the first dual-target inhibitor that has been approved for R/R DLBCL treatment in clinical trials. Its phase I study shows fimepinostat has better tolerability, and lower toxicity than other FDA approved single target HDAC or PI3K inhibitor (Younes et al., 2016). Now the efficacy is evaluating in phase II clinical trial (NCT02909777).

### Perspective

Single agents of HDACIs has low response in all types of BCL. However, the significantly synergizing effect with other drugs makes it worth to be developed, especially selective HDAC inhibitors. Class I HDAC inhibitor improved the tolerability and reduced the toxicity and HDAC6 inhibitor also showed promising effect in combination with other drugs even overcome the drug resistance. These results may encourage us to fully understand the mechanism and develop more specific selective HDACIs.

Furthermore, fimepinostat, the first-in-class dual target inhibitor for DLBCL therapies, successfully decrease the toxicity comparing to dose single target agent. This may also give us an inspiration to develop other dual-target inhibitor.

## Hodgkin Lymphomas

### Introduction

One of the most curable cancer types, Hodgkin lymphoma (HL) is a type of B cell lymphoma, which has specific and unique characteristics. Hodgkin lymphoma was first been identified by Hodgkin in 1832 (Hodgkin, 1832). Subsequently, this lymphoma was named after him in 1865 by Wilks (Wilks, 1856). According to World Health organization (WHO), HL can be classified into two main types, classical Hodgkin lymphoma (cHL) and nodular lymphocyte-predominant Hodgkin lymphoma (NLPHL). cHL accounts for 95% of all HL patients and the remainder are NLPHL. In this review we will mainly focus on cHL (Stathis and Younes, 2015).

In cHL patients, mononuclear Hodgkin cells and multinucleated Reed-Stemberg (HRS) cells arise from monoclonal B lymphocytes in the germinal center of lymphoid tissue and effect the rearrangement of IgG genes (Diehl, 2007). According to statistics, around 40% of cHL patients are infected by Epstein-Barr virus (EBV) and 100% of patients are infected with the human immunodeficiency virus (HIV). The apoptosis of these abnormal cells was inhibited in a manner which correlates with the expression of NF-κB, Notch 1 and some other transcription factors (Re et al., 2005). Research shows that, CD30 surface receptors, a member of tumor necrosis factor (TNF) receptor superfamily, will be characteristically expressed in HRS cells. The expression of TNF receptors mediates various signaling pathways, including the activation of NF-κB (Dürkop et al., 1992; Duckett et al., 1997).

To date, around 80% of cHL patients can be cured after receiving radiation therapy and chemotherapy. Recently, early and advanced stages in cHL patients were treated with doxorubicin, bleomycin, vinblastine, and dacarbazine (ABVD) in a first line and combined with bleomycin, etoposide, adriamycin, cyclophosphamide, vincristine, procarbazine, and prednisone (BEACOPP) in a second line chemotherapy regimen. Although BEACOPP showed better overall survival rate than ABVD, its high acute toxicity makes ABVD more acceptable (Carde et al., 2016). Targeting CD30 on the other hand, is another strategy for cHL. In a combination with brentuximab vedotin and ABVD, it showed a promising therapeutic effect, but resulted in high pulmonary toxicity. By omitting bleomycin the toxicity was dramatically reduced, and this type of B-AVD therapy has become popular (Younes et al., 2013). For relapsed and refractory cHL patients, platinum- or gemcitabine-based therapies were used in a first line followed by autologous stem-cell transplantation, which can cure 60% of R/R cHL patients (Clavio et al., 2005). Nowadays, cHL is almost a curable disease, but delayed treatment-related toxicity may lead to second malignancies and cardiovascular disease (Armitage, 2010), and this has inspired a search for new therapeutic strategies.

### Evidence of HDAC in Classical Hodgkin Lymphoma

As was mentioned previously, expression of abnormal HDACs has been found in both t cell and B cell lymphomas, which made it a promising therapeutic target. Because cHL is a type of B cell lymphoma, HDACs also are overexpressed in cHL. Research from Tzankov et al. shows that HDAC1, 2, and 3 are highly expressed in HRS cells. Interestingly, after treatment with HDACIs, the inhibition of HDAC1 inhibition in HRS cells leads to a poorer prognostic effect, for reasons that are still under investigation. Notwithstanding this, HDAC is still deemed a potent therapeutic target for cHL (Adams et al., 2010).

### Application of HDACIs in Classical Hodgkin Lymphomas

Recently, several HDACIs Figure 2 have been tested against R/R cHL in clinical trials, including panobinostat (Younes et al., 2012), vorinostat (NCT00132028), givinostat (NCT00496431), resminostat (NCT01037478), mocetinostat (NCT00358982), abexinostat (NCT00724984, NCT01149668), ricolinostat (NCT02091063), entinostat (NCT00866333), and tinostamustine (NCT02576496). These HDACIs were used as single treatment which brought patients positive results. Comparison, however, with other target therapies, such as PD-1 antibodies or some immunomodulatory antibodies, showed that HDACIs give relatively low overall response rates and comparable progression-free survivals (Adams et al., 2010). Above all, HDACIs might not be suitable for cHL treatment. On the other hand, HDACIs have been reported to have the ability to alter cytokines, which may enhance the immune response (Oki et al., 2014). Downregulation of PD1 on t cell and upregulation of OX40 ligand in HRS cells can exhibit antitumor immunity through HDAC11 inhibition (Buglio et al., 2011). This makes HDAC a favorable enhancer in numerous combination therapies and a number of clinical trials are now in progress. For instance, panobinostat has been used with everolimus (NCT00918333), lenalidomide (NCT01460940), cytarabine (NCT01321346), and ICE (NCT01169636). Other HDACIs are also being tested in combination therapies, such as combination of vorinostat with lenalidomide (NCT01116154), alisertib (NCT01567709), R-CHOP (NCT00667615), or a combination of mocetinostat with azacitidine (NCT00543582) and brentuximab vedotin (NCT02429375). Although some preliminary results showed high efficacy, further evaluation is necessary.

### Perspective

Similar to the role of HDACIs in BCL therapies, HDACIs are more like an enhancer in cHL therapies. It is certainly single dosage of HDACIs provided some positive result. However, other target therapies exhibited more potent in cHL therapies. In spite of that, HDACIs displayed dramatically increase of efficacy, when combined with other cHL therapies. Though, the clinical studies haven’t been completed yet, still give us some inspiration of HDACIs‘ character in cHL therapies.

## Tumor Immunity Reduction

Tumor immunity escape is an important issue in cancer therapy. As cancer cells are known to be abnormal proliferating cells, they have a unique microenvironment with which they can evade the immune system. The programmed death ligand-1/programmed death-1 (PD-L1/PD-1) pathway, is at the root of the cancer cells’ tolerance to the immune system. PD-1 overexpression in the tumor microenvironment causes an immunosuppressive effect (He et al., 2015; Jiang et al., 2019). Research on melanoma tumor cells has found that the tumor immunity is utilized by the host immune system, instead of by the tumor itself, which means that one can reduce the tumor immunity by modulating the immune system. B7 is a protein family that is found on antigen presenting cells (APC), and can bind to t cells. B7-H1, one of the B7 family members also known as PD-L1, has been found to be abundant in cancer cells. PD-L1 can be induced by cytokines such as IFN-γ or IL-8, and these cytokines are produced by CD8 + t cells, and the tumor microenvironment can be considered as a pro-inflammatory condition. Thus, targeting CD8 + t cell could be a solution to reduction of tumor immunity (Dong and Chen, 2003; Harlin et al., 2009; Spranger et al., 2013).

### Application of HDACIs to Tumor Immunity Reduction

As mentioned previously, HDACs play a crucial role in t cell regulation. In CD8 + t cells, the expression of PD1/PD-L1 has been shown through an inhibition assay by ACY241, a selective HDAC6 inhibitor to be positively controlled by HDAC6. Notably, HDACs other than HDAC6 are important in the CD8 + t cell immune response pathway (Yu et al., 2002; Adcock, 2007). Therefore, several HDACIs have been examined for their ability to reduce the tumor immunity (Bae et al., 2018).

Vorinostat and panobinostat have been used with immune cell stimulating antibodies in renal and colon carcinomas, and showed a surprising effect in inhibition of tumor growth (Christiansen et al., 2011). The selective HDAC inhibitor, entinostat, combined with IL-2 is also effective in a renal cell carcinoma mice model (Kato et al., 2007). Besides conventional HDAC inhibitors, some new compounds were synthesized for this kind of HDAC mediated immunotherapy (Vo et al., 2009). A novel HDAC and HSP90 dual inhibitor also causes downregulation of PD-L1 expression (Mehndiratta et al., 2020). These results are still in pre-clinical stages, but they provide a new aspect in immunotherapies.

## Conclusion

Lymphomas are a group of hematopoietic malignancies with complex pathogenesis and which easily relapse. Thus, new therapeutic targets are necessary. As HDAC being an important character in epigenetic modulation, HDACIs have been approved for the treatment of several cancers. These HDACIs also show high potency in treatment of lymphomas. In T cell lymphoma, single usage of HDACIs shows promising results and combination of HDACIs with conventional chemotherapies showed a synergistic effect comparable to that from a single dosage. However, in B cell lymphoma and Hodgkin lymphoma, single usage of HDACIs, shows low overall response rates, which means it may be unsuitable for both B cell lymphoma and Hodgkin lymphoma. Fortunately, when combined with chemotherapies or other targeting therapies, the therapeutic effect was surprisingly enhanced. In some of the cases, the overall response rate was increased to more than 80%. This result inspired us to focus on the character of HDACIs in combination therapies. We concluded that in studies of the role of HDACs in T cells and tumor immunity expression, HDACIs might act as an enhancer, which can reduce the tumor immunity, thereby increasing the drugs’ therapeutic effects. According to the information gained from B cell lymphoma and Hodgkin lymphoma treatments, we predict that HDACIs can not only arrest the cell cycle and trigger apoptosis, but modulate the t cell function in order to reduce tumor immunity. Indeed, there is some research in this area but still the precise mechanism should be clarified. Noticeably, HDACIs can both reduce the production of cytokines and lower the expression of PD-1. The modulation of HDACs by these two actions is already known, but their influence on the therapeutic effects remains unknown and further investigation is needed. HDACIs are still potent and prospectively useful either in immunotherapies or target therapies. We hope that HDACIs may lead us to cures for cancer in the future.

## Author Contributions

J-PL: framework design and concept development. I-CC: article composition. BS: collection. All authors contributed to the article and approved the submitted version.

## Conflict of Interest

The authors declare that the research was conducted in the absence of any commercial or financial relationships that could be construed as a potential conflict of interest.

## References

[B1] Adams H. Fritzsche F. R. Dirnhofer S. Kristiansen G. Tzankov A. (2010). Class I histone deacetylases 1, 2 and 3 are highly expressed in classical Hodgkin’s lymphoma. *Expert Opin. Ther. Targets* 14 577–584. 10.1517/14728221003796609 20415600

[B2] Adcock I. M. (2007). HDAC inhibitors as anti-inflammatory agents. *Br. J. Pharmacol.* 150 829–831. 10.1038/sj.bjp.0707166 17325655 PMC2013887

[B3] Amengual J. E. Prabhu S. A. Lombardo M. Zullo K. Johannet P. M. Gonzalez Y. (2017). Mechanisms of acquired drug resistance to the HDAC6 selective inhibitor ricolinostat reveals rational drug-drug combination with ibrutinib. *Clin. Cancer Res.* 23 3084–3096. 10.1158/1078-0432.CCR-16-2022 27993968 PMC5474138

[B4] Armitage J. O. (2010). Early-stage Hodgkin’s lymphoma. *N. Engl. J. Med.* 363 653–662. 10.1056/NEJMra1003733 20818856

[B5] Atadja P. (2009). Development of the pan-DAC inhibitor panobinostat (LBH589): successes and challenges. *Cancer Lett.* 280 233–241. 10.1016/j.canlet.2009.02.019 19344997

[B6] Azagra A. Román-González L. Collazo O. Rodríguez-Ubreva J. de Yébenes V. G. Barneda-Zahonero B. (2016). In vivo conditional deletion of HDAC7 reveals its requirement to establish proper B lymphocyte identity and development. *J. Exp. Med.* 213 2591–2601. 10.1084/jem.20150821 27810920 PMC5110011

[B7] Bae J. Hideshima T. Tai Y. T. Song Y. Richardson P. Raje N. (2018). Histone deacetylase (HDAC) inhibitor ACY241 enhances anti-tumor activities of antigen-specific central memory cytotoxic t lymphocytes against multiple myeloma and solid tumors. *Leukemia* 32 1932–1947. 10.1038/s41375-018-0062-8 29487385 PMC6537609

[B8] Barbarotta L. Hurley K. (2015). Romidepsin for the treatment of peripheral T-cell lymphoma. *J. Adv. Pract. Oncol.* 6 22–36. 26413372 PMC4577031

[B9] Barneda-Zahonero B. Collazo O. Azagra A. Fernández-Duran I. Serra-Musach J. Islam A. B. (2015). The transcriptional repressor HDAC7 promotes apoptosis and c-Myc downregulation in particular types of leukemia and lymphoma. *Cell Death Dis.* 6:e1635. 10.1038/cddis.2014.594 25675295 PMC4669785

[B10] Batlevi C. L. Crump M. Andreadis C. Rizzieri D. Assouline S. E. Fox S. (2017). A phase 2 study of mocetinostat, a histone deacetylase inhibitor, in relapsed or refractory lymphoma. *Br. J. Haematol.* 178 434–441. 10.1111/bjh.14698 28440559 PMC5576135

[B11] Berger S. (2007). The complex language of chromatin regulation during transcription. *Nature* 447 407–412. 10.1038/nature05915 17522673

[B12] Boucheron N. Tschismarov R. Goeschl L. Moser M. A. Lagger S. Sakaguchi S. (2014). CD4+ t cell lineage integrity is controlled by the histone deacetylases HDAC1 and HDAC2. *Nat. Immunol.* 15 439–448. 10.1038/ni.2864 24681565 PMC4346201

[B13] Boumber Y. Younes A. Garcia-Manero G. (2011). Mocetinostat (MGCD0103): a review of an isotype-specific histone deacetylase inhibitor. *Expert Opin. Investig. Drugs* 20 823–829. 10.1517/13543784.2011.577737 21554162 PMC5206967

[B14] Budde L. E. Zhang M. M. Shustov A. R. Pagel J. M. Gooley T. A. Oliveira G. R. (2013). A phase I study of pulse high-dose vorinostat (V) plus rituximab (R), ifosphamide, carboplatin, and etoposide (ICE) in patients with relapsed lymphoma. *Br. J. Haematol.* 161 183–191. 10.1111/bjh.12230 23356514 PMC3618618

[B15] Buglio D. Khaskhely N. M. Voo K. S. Martinez-Valdez H. Liu Y. J. Younes A. (2011). HDAC11 plays an essential role in regulating OX40 ligand expression in Hodgkin lymphoma. *Blood* 117 2910–2917. 10.1182/blood-2010-08-303701 21239696 PMC3062301

[B16] Campbell P. Thomas C. M. (2017). Belinostat for the treatment of relapsed or refractory peripheral T-cell lymphoma. *J. Oncol. Pharm. Pract.* 23 143–147. 10.1177/1078155216634178 26921086

[B17] Carde P. Karrasch M. Fortpied C. Brice P. Khaled H. Casasnovas O. (2016). Eight cycles of ABVD versus four cycles of BEACOPPescalated plus four cycles of BEACOPPbaseline in stage III to IV, international prognostic score = 3, high-risk Hodgkin lymphoma: first results of the phase III EORTC 20012 intergroup trial. *J. Clin. Oncol.* 34 2028–2036. 10.1200/JCO.2015.64.5648 27114593

[B18] Chen R. Frankel P. Popplewell L. Siddiqi T. Ruel N. Rotter A. (2015). A phase II study of vorinostat and rituximab for treatment of newly diagnosed and relapsed/refractory indolent non-Hodgkin lymphoma. *Haematologica* 100 357–362. 10.3324/haematol.2014.117473 25596263 PMC4349274

[B19] Cheng F. Lienlaf M. Wang H. W. Perez-Villarroel P. Lee C. Woan K. (2014). A novel role for histone deacetylase 6 in the regulation of the tolerogenic STAT3/IL-10 pathway in APCs. *J. Immunol.* 193 2850–2862. 10.4049/jimmunol.1302778 25108026 PMC4157123

[B20] Child F. Ortiz-Romero P. L. Alvarez R. Bagot M. Stadler R. Weichenthal M. (2016). Phase II multicentre trial of oral quisinostat, a histone deacetylase inhibitor, in patients with previously treated stage IB-IVA mycosis fungoides/Sézary syndrome. *Br. J. Dermatol.* 175 80–88. 10.1111/bjd.14427 26836950

[B21] Choudhary C. Kumar C. Gnad F. Nielsen M. L. Rehman M. Walther T. C. (2009). Lysine acetylation targets protein complexes and co-regulates major cellular functions. *Science* 325 834–840. 10.1126/science.1175371 19608861

[B22] Chowdhury S. Howell G. M. Teggart C. A. Chowdhury A. Person J. J. Bowers D. M. (2011). Histone deacetylase inhibitor belinostat represses survivin expression through reactivation of transforming growth factor beta (TGFbeta) receptor II leading to cancer cell death. *J. Biol. Chem.* 286 30937–30948. 10.1074/jbc.M110.212035 21757750 PMC3162453

[B23] Christiansen A. J. West A. Banks K. M. Haynes N. M. Teng M. W. Smyth M. J. (2011). Eradication of solid tumors using histone deacetylase inhibitors combined with immune-stimulating antibodies. *Proc. Natl. Acad. Sci. U.S.A.* 108 4141–4146. 10.1073/pnas.1011037108 21368108 PMC3054015

[B24] Clavio M. Garrone A. Pierri I. Michelis G. L. Balocco M. Albarello A. (2005). Ifosfamide, epirubicin, etoposide (IEV) and autologous peripheral blood progenitor cell transplant: a feasible and effective salvage treatment for lymphoid malignancies. *Oncol. Rep.* 14 933–940. 16142354

[B25] Cosenza M. Civallero M. Fiorcari S. Pozzi S. Marcheselli L. Bari A. (2016). The histone deacetylase inhibitor romidepsin synergizes with lenalidomide and enhances tumor cell death in T-cell lymphoma cell lines. *Cancer Biol. Ther.* 17 1094–1106. 10.1080/15384047.2016.1219820 27657380 PMC5079402

[B26] Cosenza M. Civallero M. Marcheselli L. Sacchi S. Pozzi S. (2017). Ricolinostat, a selective HDAC6 inhibitor, shows anti-lymphoma cell activity alone and in combination with bendamustine. *Apoptosis* 22 827–840. 10.1007/s10495-017-1364-4 28315173 PMC5401712

[B27] Crump M. Coiffier B. Jacobsen E. D. Sun L. Ricker J. L. Xie H. (2008). Phase II trial of oral vorinostat (suberoylanilide hydroxamic acid) in relapsed diffuse large-B-cell lymphoma. *Ann. Oncol.* 19 964–969. 10.1093/annonc/mdn031 18296419

[B28] Dahiya S. Beier U. H. Wang L. Han R. Jiao J. Akimova T. (2020). HDAC10 deletion promotes Foxp3+ T-regulatory cell function. *Sci. Rep.* 10:424. 10.1038/s41598-019-57294-x 31949209 PMC6965082

[B29] Dasmahapatra G. Patel H. Friedberg J. Quayle S. N. Jones S. S. Grant S. (2014). In vitro and in vivo interactions between the HDAC6 inhibitor ricolinostat (ACY1215) and the irreversible proteasome inhibitor carfilzomib in non-Hodgkin lymphoma cells. *Mol. Cancer Ther.* 13 2886–2897. 10.1158/1535-7163.MCT-14-0220 25239935 PMC4304772

[B30] Dequiedt F. Kasler H. Fischle W. Kiermer V. Weinstein M. Herndier B. G. (2003). HDAC7, a thymus-specific class II histone deacetylase, regulates Nur77 transcription and TCR-mediated apoptosis. *Immunity* 18 687–698. 10.1016/S1074-7613(03)00109-2 12753745

[B31] Diehl V. (2007). Hodgkin’s disease–from pathology specimen to cure. *N. Engl. J. Med.* 357 1968–1971. 10.1056/NEJMe078173 17989391

[B32] Ding H. Peterson K. L. Correia C. Koh B. Schneider P. A. Nowakowski G. S. (2017). Histone deacetylase inhibitors interrupt HSP90RASGRP1 and HSP90CRAF interactions to upregulate BIM and circumvent drug resistance in lymphoma cells. *Leukemia* 31 1593–1602. 10.1038/leu.2016.357 27890930 PMC5474223

[B33] Dong H. Chen L. (2003). B7-H1 pathway and its role in the evasion of tumor immunity. *J. Mol. Med.* 81 281–287. 10.1007/s00109-003-0430-2 12721664

[B34] Dovey O. M. Foster C. T. Conte N. Edwards S. A. Edwards J. M. Singh R. (2013). Histone deacetylase 1 and 2 are essential for normal T-cell development and genomic stability in mice. *Blood* 121 1335–1344. 10.1182/blood-2012-07-441949 23287868 PMC3836254

[B35] Duckett C. S. Gedrich R. W. Gilfillan M. C. Thompson C. B. (1997). Induction of nuclear factor kappaB by the CD30 receptor is mediated by TRAF1 and TRAF2. *Mol. Cell Biol.* 17 1535–1542. 10.1128/mcb.17.3.1535 9032281 PMC231879

[B36] Dupuis J. Morschhauser F. Ghesquières H. Tilly H. Casasnovas O. Thieblemont C. (2015). Combination of romidepsin with cyclophosphamide, doxorubicin, vincristine, and prednisone in previously untreated patients with peripheral T-cell lymphoma: a non-randomised, phase 1b/2 study. *Lancet Haematol.* 2 e160–e165. 10.1016/S2352-3026(15)00023-X 26687958

[B37] Dürkop H. Latza U. Hummel M. Eitelbach F. Seed B. Stein H. (1992). Molecular cloning and expression of a new member of the nerve growth factor receptor family that is characteristic for Hodgkin’s disease. *Cell* 68 421–427. 10.1016/0092-8674(92)90180-k 1310894

[B38] Duvic M. Dummer R. Becker J. C. Poulalhon N. Romero P. O. Bernengo M. G. (2013). Panobinostat activity in both bexarotene-exposed and -naïve patients with refractory cutaneous T-cell lymphoma: results of a phase II trial. *Eur. J. Cancer* 49 386–394. 10.1016/j.ejca.2012.08.017 22981498

[B39] Duvic M. Talpur R. Ni X. Zhang C. Hazarika P. Kelly C. (2007). Phase 2 trial of oral vorinostat (suberoylanilide hydroxamic acid, SAHA) for refractory cutaneous T-cell lymphoma (CTCL). *Blood* 109 31–39. 10.1182/blood-2006-06-025999 16960145 PMC1785068

[B40] Duvic M. Vu J. (2007). Vorinostat: a new oral histone deacetylase inhibitor approved for cutaneous T-cell lymphoma. *Expert Opin. Investig. Drugs* 16 1111–1120. 10.1517/13543784.16.7.1111 17594194

[B41] Ellis L. Pan Y. Smyth G. K. George D. J. McCormack C. Williams-Truax R. (2008). Histone deacetylase inhibitor panobinostat induces clinical responses with associated alterations in gene expression profiles in cutaneous T-cell lymphoma. *Clin. Cancer Res.* 14 4500–4510. 10.1158/1078-0432.ccr-07-4262 18628465

[B42] Ellmeier W. (2015). Molecular control of CD4(+) t cell lineage plasticity and integrity. *Int. Immunopharmacol.* 28 813–817. 10.1016/j.intimp.2015.03.050 25864621

[B43] Eyre T. A. Collins G. P. Gupta A. Coupe N. Sheikh S. Whittaker J. (2019). A phase 1 study to assess the safety, tolerability, and pharmacokinetics of CXD101 in patients with advanced cancer. *Cancer* 125 99–108. 10.1002/cncr.31791 30332497

[B44] Foss F. Pro B. Miles Prince H. Sokol L. Caballero D. Horwitz S. (2017). Responses to romidepsin by line of therapy in patients with relapsed or refractory peripheral T-cell lymphoma. *Cancer Med.* 6 36–44. 10.1002/cam4.939 27981793 PMC5269566

[B45] Gammoh N. Lam D. Puente C. Ganley I. Marks P. A. Jiang X. (2006). Role of autophagy in histone deacetylase inhibitor-induced apoptotic and nonapoptotic cell death. *Proc. Natl. Acad. Sci. U.S.A.* 109 6561–6565. 10.1073/pnas.1204429109 22493260 PMC3340088

[B46] Gil V. S. Bhagat G. Howell L. Zhang J. Kim C. H. Stengel S. (2016). Deregulated expression of HDAC9 in B cells promotes development of lymphoproliferative disease and lymphoma in mice. *Dis. Model Mech.* 9 1483–1495. 10.1242/dmm.023366 27799148 PMC5200892

[B47] Harlin H. Meng Y. Peterson A. C. Zha Y. Tretiakova M. Slingluff C. (2009). Chemokine expression in melanoma metastases associated with CD8+ T-cell recruitment. *Cancer Res.* 69 3077–3085. 10.1158/0008-5472.CAN-08-2281 19293190 PMC3886718

[B48] He J. Hu Y. Hu M. Li B. (2015). Development of PD-1/PD-L1 pathway in tumor immune microenvironment and treatment for non-small cell lung cancer. *Sci. Rep.* 5:13110. 10.1038/srep13110 26279307 PMC4538573

[B49] Heider U. Rademacher J. Lamottke B. Mieth M. Moebs M. von Metzler I. (2009). Synergistic interaction of the histone deacetylase inhibitor SAHA with the proteasome inhibitor bortezomib in cutaneous t cell lymphoma. *Eur. J. Haematol.* 82 440–449. 10.1111/j.1600-0609.2009.01239.x 19220424

[B50] Hodgkin T. (1832). On some morbid appearances of the absorbent glands and spleen. *Med. Chir. Trans.* 17 68–114. 10.1177/095952873201700106 20895597 PMC2116706

[B51] Hopfinger G. Nösslinger T. Lang A. Linkesch W. Melchardt T. Weiss L. (2014). Lenalidomide in combination with vorinostat and dexamethasone for the treatment of relapsed/refractory peripheral t cell lymphoma (PTCL): report of a phase I/II trial. *Ann. Hematol.* 93 459–462. 10.1007/s00277-014-2009-0 24441915

[B52] Hsu F. C. Belmonte P. J. Constans M. M. Chen M. W. McWilliams D. C. Hiebert S. W. (2015). Histone deacetylase 3 is required for t cell maturation. *J. Immunol.* 195 1578–1590. 10.4049/jimmunol.1500435 26163592 PMC4530026

[B53] Huang J. Wang L. Dahiya S. Beier U. H. Han R. Samanta A. (2017). Histone/protein deacetylase 11 targeting promotes Foxp3+ Treg function. *Sci. Rep.* 7:8626. 10.1038/s41598-017-09211-3 PMC5561267 28819166

[B54] Hull E. E. Montgomery M. R. Leyva K. J. (2016). HDAC inhibitors as epigenetic regulators of the immune system: impacts on cancer therapy and inflammatory diseases. *Biomed Res. Int.* 2016:8797206. 10.1155/2016/8797206 27556043 PMC4983322

[B55] Hutt D. M. Roth D. M. Vignaud H. Cullin C. Bouchecareilh M. (2014). The histone deacetylase inhibitor, Vorinostat, represses hypoxia inducible factor 1 alpha expression through translational inhibition. *PLoS One* 9:e106224. 10.1371/journal.pone.0106224 25166596 PMC4148404

[B56] Imam M. H. Shenoy P. J. Flowers C. R. Phillips A. Lechowicz M. J. (2013). Incidence and survival patterns of cutaneous T-cell lymphomas in the United States. *Leuk. Lymphoma* 54 752–759. 10.3109/10428194.2012.729831 23004352

[B57] Irlé C. Weintraub J. (2016). Long-term treatment with romidepsin in patients with peripheral T-cell lymphoma. *Case Rep. Hematol.* 2016:8175957. 10.1155/2016/8175957 27648317 PMC5014944

[B58] Iyer S. P. Foss F. F. (2015). Romidepsin for the treatment of peripheral T-cell lymphoma. *Oncologist* 20 1084–1091. 10.1634/theoncologist.2015-0043 26099743 PMC4571813

[B59] Jenuwein T. Allis C. D. (2001). Translating the histone code. *Science* 293 1074–1080. 10.1126/science.1063127 11498575

[B60] Jiang X. Wang J. Deng X. Xiong F. Ge J. Xiang B. (2019). Role of the tumor microenvironment in PD-L1/PD-1-mediated tumor immune escape. *Mol. Cancer.* 18:10. 10.1186/s12943-018-0928-4 30646912 PMC6332843

[B61] Jiang Y. Ortega-Molina A. Geng H. Ying H. Y. Hatzi K. Parsa S. (2017). CREBBP inactivation promotes the development of HDAC3-dependent lymphomas. *Cancer Discov.* 7 38–53. 10.1158/2159-8290.CD-16-0975 27733359 PMC5300005

[B62] Johnston P. B. Cashen A. F. Nikolinakos P. G. Beaven A. W. Barta S. K. Bhat G. (2015). Safe and effective treatment of patients with peripheral T-cell lymphoma (PTCL) with the novel HDAC inhibitor, belinostat, in combination with CHOP: results of the bel-CHOP phase 1 trial. *Blood* 126:253 10.1182/blood.V126.23.253.253

[B63] Kato Y. Yoshimura K. Shin T. Verheul H. Hammers H. Sanni T. B. (2007). Synergistic in vivo antitumor effect of the histone deacetylase inhibitor MS-275 in combination with interleukin 2 in a murine model of renal cell carcinoma. *Clin. Cancer Res.* 13 4538–4546. 10.1158/1078-0432.CCR-07-0014 17671140

[B64] Kirschbaum M. Frankel P. Popplewell L. Zain J. Delioukina M. Pullarkat V. (2011). Phase II study of vorinostat for treatment of relapsed or refractory indolent non-Hodgkin’s lymphoma and mantle cell lymphoma. *J. Clin. Oncol.* 29 1198–1203. 10.1200/JCO.2010.32.1398 21300924 PMC3083875

[B65] Kunami N. Katsuya H. Nogami R. Ishitsuka K. Tamura K. (2014). Promise of combining a Bcl-2 family inhibitor with bortezomib or SAHA for adult T-cell leukemia/lymphoma. *Anticancer Res.* 34 5287–5294. 25275021

[B66] Lemercier C. Brocard M. P. Puvion-Dutilleul F. Kao H. Y. Albagli O. Khochbin S. (2002). Class II histone deacetylases are directly recruited by BCL6 transcriptional repressor. *J. Biol. Chem.* 277 22045–22052. 10.1074/jbc.M201736200 11929873

[B67] Li Y. Seto E. (2016). HDACs and HDAC inhibitors in cancer development and therapy. *Cold Spring Harb. Perspect. Med.* 6:a026831. 10.1101/cshperspect.a026831 27599530 PMC5046688

[B68] Liu Z. Cai Y. Yang Y. Li A. Bi R. Wang L. (2018). Activation of MET signaling by HDAC6 offers a rationale for a novel ricolinostat and crizotinib combinatorial therapeutic strategy in diffuse large B-cell lymphoma. *J. Pathol.* 246 141–153. 10.1002/path.5108 29876933

[B69] Loosveld M. Castellano R. Gon S. Goubard A. Crouzet T. Pouyet L. (2014). Therapeutic targeting of c-Myc in T-cell acute lymphoblastic leukemia, T-ALL. *Oncotarget* 30 3168–3172. 10.18632/oncotarget.1873 24930440 PMC4102800

[B70] Lu X. Ning Z. Li Z. Cao H. Wang X. (2016). Development of chidamide for peripheral T-cell lymphoma, the first orphan drug approved in China. *Intractable Rare Dis. Res.* 5 185–191. 10.5582/irdr.2016.01024 27672541 PMC4995415

[B71] Martin M. Potente M. Janssens V. Vertommen D. Twizere J. C. Rider M. H. (2008). Protein phosphatase 2A controls the activity of histone deacetylase 7 during t cell apoptosis and angiogenesis. *Proc. Natl. Acad. Sci. U.S.A.* 105 4727–4732. 10.1073/pnas.0708455105 18339811 PMC2290748

[B72] McCann S. A. Story S. K. (2013). Histone deacetylase inhibitors in cutaneous T-cell lymphoma. *J. Dermatol. Nurs. Assoc.* 5 305–313. 10.1097/JDN.0000000000000007

[B73] Mehndiratta S. Lin M. H. Wu Y. W. Chen C. H. Wu T. Y. Chuang K. H. (2020). N-alkyl-hydroxybenzoyl anilide hydroxamates as dual inhibitors of HDAC and HSP90, downregulating IFN-γ induced PD-L1 expression. *Eur. J. Med. Chem.* 185:111725. 10.1016/j.ejmech.2019.111725 31655430

[B74] Mishra A. La Perle K. Kwiatkowski S. Sullivan L. A. Sams G. H. Johns J. (2016). Mechanism, consequences, and therapeutic targeting of abnormal IL15 signaling in cutaneous T-cell lymphoma. *Cancer Discov.* 6 986–1005. 10.1158/2159-8290 27422033 PMC5388135

[B75] Molecule of the month (2006). Molecule of the month. *Vorinostat. Drug News Perspect.* 19:352. 16971971

[B76] Nanou A. Toumpeki C. Lavigne M. D. Lazou V. Demmers J. Paparountas T. (2017). The dual role of LSD1 and HDAC3 in STAT5-dependent transcription is determined by protein interactions, binding affinities, motifs and genomic positions. *Nucleic Acids Res.* 45 142–154. 10.1093/nar/gkw832 27651463 PMC5224505

[B77] Nieto Y. Valdez B. C. Thall P. F. Ahmed S. Jones R. B. Hosing C. (2015). Vorinostat combined with high-dose gemcitabine, busulfan, and melphalan with autologous stem cell transplantation in patients with refractory lymphomas. *Biol. Blood Marrow Transplant.* 21 1914–1920. 10.1016/j.bbmt.2015.06.003 26071868 PMC4781754

[B78] Nieto Y. Valdez B. C. Thall P. F. Jones R. B. Wei W. Myers A. (2016). Double epigenetic modulation of high-dose chemotherapy with azacitidine and vorinostat for patients with refractory or poor-risk relapsed lymphoma. *Cancer* 122 2680–2688. 10.1002/cncr.30100 27203405 PMC4992444

[B79] Ning Z. Q. Li Z. B. Newman M. J. Shan S. Wang X. H. Pan D. S. (2012). Chidamide (CS055/HBI-8000): a new histone deacetylase inhibitor of the benzamide class with antitumor activity and the ability to enhance immune cell-mediated tumor cell cytotoxicity. *Cancer Chemother. Pharmacol.* 69 901–909. 10.1007/s00280-011-1766-x 22080169

[B80] Nishioka C. Ikezoe T. Yang J. Komatsu N. Bandobashi K. Taniguchi A. (2008). Histone deacetylase inhibitors induce growth arrest and apoptosis of HTLV-1-infected T-cells via blockade of signaling by nuclear factor κB. *Leuk. Res.* 32 287–296. 10.1016/j.leukres.2007.05.026 17644177

[B81] Noureen N. Rashid H. Kalsoom S. (2010). Identification of type-specific anticancer histone deacetylase inhibitors: road to success. *Cancer Chemother. Pharmacol.* 66 625–633. 10.1007/s00280-010-1324-y 20401613

[B82] Ogura M. Ando K. Suzuki T. Ishizawa K. Oh S. Y. Itoh K. (2014). A multicentre phase II study of vorinostat in patients with relapsed or refractory indolent B-cell non-Hodgkin lymphoma and mantle cell lymphoma. *Br. J. Haematol.* 165 768–776. 10.1111/bjh.12819 24617454 PMC4282031

[B83] Oki Y. Buglio D. Fanale M. Fayad L. Copeland A. Romaguera J. (2013a). Phase I study of panobinostat plus everolimus in patients with relapsed or refractory lymphoma. *Clin. Cancer Res.* 19 6882–6890. 10.1158/1078-0432.CCR-13-1906 24097867 PMC4409814

[B84] Oki Y. Younes A. Copeland A. Hagemeister F. Fayad L. E. McLaughlin P. (2013b). Phase I study of vorinostat in combination with standard CHOP in patients with newly diagnosed peripheral T-cell lymphoma. *Br. J. Haematol.* 162 138–141. 10.1111/bjh.12326 23590726

[B85] Oki Y. Buglio D. Zhang J. Ying Y. Zhou S. Sureda A. (2014). Immune regulatory effects of panobinostat in patients with Hodgkin lymphoma through modulation of serum cytokine levels and T-cell PD1 expression. *Blood Cancer J.* 4:e236. 10.1038/bcj.2014.58 25105535 PMC4219471

[B86] Olsen E. A. Kim Y. H. Kuzel T. M. Pacheco T. R. Foss F. M. Parker S. (2007). Phase IIB multicenter trial of vorinostat in patients with persistent, progressive, or treatment refractory cutaneous T-cell lymphoma. *J. Clin. Oncol.* 25 3109–3115. 10.1200/JCO.2006.10.243 17577020

[B87] Palermo R. Checquolo S. Giovenco A. Grazioli P. Kumar V. Campese A. F. (2012). Acetylation controls Notch3 stability and function in T-cell leukemia. *Oncogene* 31 3807–3817. 10.1038/onc.2011.533 22120716

[B88] Pasqualucci L. Dominguez-Sola D. Chiarenza A. Fabbri G. Grunn A. Trifonov V. (2011). Inactivating mutations of acetyltransferase genes in B-cell lymphoma. *Nature* 471 189–195. 10.1038/nature09730 21390126 PMC3271441

[B89] Persky D. O. Li H. Rimsza L. M. Barr P. M. Popplewell L. L. Bane C. L. (2018). A phase I/II trial of vorinostat (SAHA) in combination with rituximab-CHOP in patients with newly diagnosed advanced stage diffuse large B-cell lymphoma (DLBCL): SWOG S0806. *Am. J. Hematol.* 93 486–493. 10.1002/ajh.25010 29266344 PMC5842116

[B90] Philips R. L. Chen M. W. McWilliams D. C. Belmonte P. J. Constans M. M. Shapiro V. S. (2016). HDAC3 is required for the downregulation of RORγt during thymocyte positive selection. *J. Immunol.* 197 541–554. 10.4049/jimmunol.1502529 27279370 PMC4940140

[B91] Philips R. L. Lee J. H. Gaonkar K. Chanana P. Chung J. Y. Romero Arocha S. R. (2019). HDAC3 restrains CD8-lineage genes to maintain a bi-potential state in CD4+CD8+ thymocytes for CD4-lineage commitment. *eLife* 8:e43821. 10.7554/eLife.43821 30657451 PMC6338460

[B92] Poole R. M. (2014). Belinostat: first global approval. *Drugs* 74 1543–1554. 10.1007/s40265-014-0275-8 25134672

[B93] Powers J. J. Maharaj K. K. Sahakian E. Xing L. PerezVillarroel P. Knox T. (2014). Histone deacetylase 6 (HDAC6) as a regulator of immune check-point molecules in chronic lymphocytic leukemia (CLL). *Blood* 124 3311–3311. 10.1182/blood.V124.21.3311.3311

[B94] Preglej T. Hamminger P. Luu M. Bulat T. Andersen L. Göschl L. (2020). Histone deacetylases 1 and 2 restrain CD4+ cytotoxic t lymphocyte differentiation. *JCI Insight* 5:e133393. 10.1172/jci.insight.133393 32102981 PMC7101144

[B95] Puvvada S. D. Li H. Rimsza L. M. Bernstein S. H. Fisher R. I. LeBlanc M. (2016). A phase II study of belinostat (PXD101) in relapsed and refractory aggressive B-cell lymphomas: SWOG S0520. *Leuk. Lymphoma* 57 2359–2369. 10.3109/10428194.2015.1135431 26758422 PMC5140034

[B96] Re D. Thomas R. K. Behringer K. Diehl V. (2005). From Hodgkin disease to Hodgkin lymphoma: biologic insights and therapeutic potential. *Blood* 105 4553–4560. 10.1182/blood-2004-12-4750 15728122

[B97] Reddy S. A. (2016). Romidepsin for the treatment of relapsed/refractory cutaneous T-cell lymphoma (mycosis fungoides/Sézary syndrome): use in a community setting. *Crit. Rev. Oncol. Hematol.* 106 99–107. 10.1016/j.critrevonc.2016.07.001 27637355

[B98] Rozati S. Cheng P. F. Widmer D. S. Fujii K. Levesque M. P. Dummer R. (2016). Romidepsin and azacitidine synergize in their epigenetic modulatory effects to induce apoptosis in CTCL. *Clin. Cancer Res.* 22 2020–2031. 10.1158/1078-0432.ccr-15-1435 26660520

[B99] Samimi S. Morrissey K. Anshelevich S. Evans K. Gardner J. Musiek A. (2013). Romidepsin and interferon gamma: a novel combination for refractory cutaneous T-cell lymphoma. *J. Am. Acad. Dermatol.* 68 e5–e6. 10.1016/j.jaad.2011.06.043 23244387

[B100] Sanaei M. Fraidoon K. (2019). Histone deacetylases and histone deacetylase inhibitors: molecular mechanisms of action in various cancers. *Adv. Biomed. Res.* 8:63 10.4103/abr.abr_142_19 PMC6839273 31737580

[B101] Sanaei M. Kavoosi F. (2019). Histone deacetylases and histone deacetylase inhibitors: molecular mechanisms of action in various cancers. *Adv. Biomed. Res.* 8:63 10.4103/abr.abr_142_19 PMC6839273 31737580

[B102] Sandhu S. K. Volinia S. Costinean S. Galasso M. Neinast R. Santhanam R. (2012). miR-155 targets histone deacetylase 4 (HDAC4) and impairs transcriptional activity of B-cell lymphoma 6 (BCL6) in the Eμ-miR-155 transgenic mouse model. *Proc. Natl. Acad. Sci. U.S.A.* 109 20047–20052. 10.1073/pnas.1213764109 23169640 PMC3523868

[B103] Seo Y. H. (2015). Dual inhibitors against topoisomerases and histone deacetylases. *J. Cancer Prev.* 20 85–91. 10.15430/JCP.2015.20.2.85 26151040 PMC4492363

[B104] Seto E. Yoshida M. (2014). Erasers of histone acetylation: the histone deacetylase enzymes. *Cold Spring Harb. Perspect. Biol.* 6:a018713. 10.1101/cshperspect.a018713 24691964 PMC3970420

[B105] Shao R. H. Tian X. Gorgun G. Urbano A. G. Foss F. M. (2002). Arginine butyrate increases the cytotoxicity of DAB389IL-2 in leukemia and lymphoma cells by upregulation of IL-2Rβ gene. *Leuk. Res.* 26 1077–1083. 10.1016/S0145-2126(02)00059-0 12443879

[B106] Spranger S. Spaapen R. M. Zha Y. Williams J. Meng Y. Ha T. T. (2013). Up-regulation of PD-L1, IDO, and T(regs) in the melanoma tumor microenvironment is driven by CD8(+) T cells. *Sci. Transl. Med.* 5:200ra116. 10.1126/scitranslmed.3006504 23986400 PMC4136707

[B107] Stathis A. Younes A. (2015). The new therapeutical scenario of Hodgkin lymphoma. *Ann. Oncol.* 26 2026–2033. 10.1093/annonc/mdv256 26037796

[B108] Stengel K. R. Barnett K. R. Wang J. Liu Q. Hodges E. Hiebert S. W. (2017). Deacetylase activity of histone deacetylase 3 is required for productive VDJ recombination and B-cell development. *Proc. Natl. Acad. Sci. U.S.A.* 114 8608–8613. 10.1073/pnas.1701610114 28739911 PMC5559004

[B109] Stengel K. R. Zhao Y. Klus N. J. Kaiser J. F. Gordy L. E. Joyce S. (2015). Histone deacetylase 3 is required for efficient T cell development. *Mol. Cell Biol.* 35 3854–3865. 10.1128/mcb.00706-15 26324326 PMC4609739

[B110] Straus D. J. Hamlin P. A. Matasar M. J. Lia Palomba M. Drullinsky P. R. Zelenetz A. D. (2015). Phase I/II trial of vorinostat with rituximab, cyclophosphamide, etoposide and prednisone as palliative treatment for elderly patients with relapsed or refractory diffuse large B-cell lymphoma not eligible for autologous stem cell transplantation. *Br. J. Haematol.* 168 663–670. 10.1111/bjh.13195 25316653

[B111] Sung J. J. Ververis K. Karagiannis T. C. (2014). Histone deacetylase inhibitors potentiate photochemotherapy in cutaneous T-cell lymphoma MyLa cells. *J. Photochem. Photobiol. B* 131 104–112. 10.1016/j.jphotobiol.2014.01.009 24518645

[B112] Tan D. Phipps C. Hwang W. Y. Tan S. Y. Yeap C. H. Chan Y. H. (2015). Panobinostat in combination with bortezomib in patients with relapsed or refractory peripheral T-cell lymphoma: an open-label, multicentre phase 2 trial. *Lancet Haematol.* 2 e326–e333. 10.1016/S2352-3026(15)00097-6 26688485

[B113] Tanaka H. Muto A. Shima H. Katoh Y. Sax N. Tajima S. (2016). Epigenetic regulation of the blimp-1 gene (Prdm1) in B cells involves Bach2 and histone deacetylase 3. *J. Biol. Chem.* 291 6316–6330. 10.1074/jbc.M116.713842 26786103 PMC4813568

[B114] Thapa P. Chen M. W. McWilliams D. C. Belmonte P. Constans M. Sant’Angelo D. B. (2016). NKAP regulates invariant NKT cell proliferation and differentiation into ROR-γt-expressing NKT17 cells. *J. Immunol.* 196 4987–4998. 10.4049/jimmunol.1501653 27183586 PMC4893932

[B115] Thapa P. Romero Arocha S. Chung J. Y. Sant’Angelo D. B. Shapiro V. S. (2017). Histone deacetylase 3 is required for iNKT cell development. *Sci. Rep.* 7:5784. 10.1038/s41598-017-06102-5 28724935 PMC5517478

[B116] Ueda H. Nakajima H. Hori Y. Goto T. Okuhara M. (1994). Action of FR901228, a novel antitumor bicyclic depsipeptide produced by *Chromobacterium violaceum* no. 968, on Ha-ras transformed NIH3T3 cells. *Biosci. Biotechnol. Biochem.* 58 1579–1583. 10.1271/bbb.58.1579 7765477

[B117] Villagra A. Cheng F. Wang H. W. Suarez I. Glozak M. Maurin M. (2009). The histone deacetylase HDAC11 regulates the expression of interleukin 10 and immune tolerance. *Nat. Immunol.* 10 92–100. 10.1038/ni.1673 19011628 PMC3925685

[B118] Vo D. D. Prins R. M. Begley J. L. Donahue T. R. Morris L. F. Bruhn K. W. (2009). Enhanced antitumor activity induced by adoptive T-cell transfer and adjunctive use of the histone deacetylase inhibitor LAQ824. *Cancer Res.* 69 8693–8699. 10.1158/0008-5472.CAN-09-1456 19861533 PMC2779578

[B119] Wang L. Qin W. Huo Y. J. Li X. Shi Q. Rasko J. (2020). Advances in targeted therapy for malignant lymphoma. *Signal Transduct. Target. Ther.* 5:15. 10.1038/s41392-020-0113-2 32296035 PMC7058622

[B120] Wang P. Wang Z. Liu J. (2020). Role of HDACs in normal and malignant hematopoiesis. *Mol. Cancer* 19:5. 10.1186/s12943-019-1127-7 31910827 PMC6945581

[B121] Wilks S. (1856). Cases of enlargement of the lymphatic glands and spleen (or, Hodgkin’s disease) with remarks. *Guys Hosp. Rep.* 11 56–67.

[B122] Wozniak M. B. Villuendas R. Bischoff J. R. Aparicio C. B. Martínez Leal J. F. de La Cueva P. (2010). Vorinostat interferes with the signaling transduction pathway of T-cell receptor and synergizes with phosphoinositide-3 kinase inhibitors in cutaneous T-cell lymphoma. *Haematologica* 95 613–621. 10.3324/haematol.2009.013870 20133897 PMC2857191

[B123] Xiao H. Jiao J. Wang L. O’Brien S. Newick K. Wang L. C. (2016). HDAC5 controls the functions of Foxp3(+) T-regulatory and CD8(+) T cells. *Int. J. Cancer* 138 2477–2486. 10.1002/ijc.29979 26704363 PMC5484398

[B124] Yamaguchi T. Cubizolles F. Zhang Y. Reichert N. Kohler H. Seiser C. (2010). Histone deacetylases 1 and 2 act in concert to promote the G1-to-S progression. *Genes Dev.* 24 455–469. 10.1101/gad.552310 20194438 PMC2827841

[B125] Yoo C. B. Jones P. A. (2006). Epigenetic therapy of cancer: past, present and future. *Nat. Rev. Drug Discov.* 5 537–550. 10.1038/nrd1930 16485345

[B126] Younes A. Berdeja J. G. Patel M. R. Flinn I. Gerecitano J. F. Neelapu S. S. (2016). Safety, tolerability, and preliminary activity of CUDC-907, a first-in-class, oral, dual inhibitor of HDAC and PI3K, in patients with relapsed or refractory lymphoma or multiple myeloma: an open-label, dose-escalation, phase 1 trial. *Lancet Oncol.* 17 622–631. 10.1016/S1470-2045(15)00584-7 27049457 PMC5494693

[B127] Younes A. Connors J. M. Park S. I. Fanale M. O’Meara M. M. Hunder N. N. (2013). Brentuximab vedotin combined with ABVD or AVD for patients with newly diagnosed Hodgkin’s lymphoma: a phase 1, open-label, dose-escalation study. *Lancet Oncol.* 14 1348–1356. 10.1016/S1470-2045(13)70501-1 24239220

[B128] Younes A. Sureda A. Ben-Yehuda D. Zinzani P. L. Ong T. C. Prince H. M. (2012). Panobinostat in patients with relapsed/refractory Hodgkin’s lymphoma after autologous stem-cell transplantation: results of a phase II study. *J. Clin. Oncol.* 30 2197–2203. 10.1200/JCO.2011.38.1350 22547596

[B129] Yu J. Angelin-Duclos C. Greenwood J. Liao J. Calame K. (2000). Transcriptional repression by blimp-1 (PRDI-BF1) involves recruitment of histone deacetylase. *Mol. Cell Biol.* 20 2592–2603. 10.1128/mcb.20.7.2592-2603.2000 10713181 PMC85475

[B130] Yu Z. Zhang W. Kone B. C. (2002). Histone deacetylases augment cytokine induction of the iNOS gene. *J. Am. Soc. Nephrol.* 13 2009–2017. 10.1097/01.ASN.0000024253.59665.F1 12138131

[B131] Zhang C. Richon V. Ni X. Talpur R. Duvic M. (2005). Selective induction of apoptosis by histone deacetylase inhibitor SAHA in cutaneous T-cell lymphoma cells: relevance to mechanism of therapeutic action. *J. Invest. Dermatol.* 125 1045–1052. 10.1111/j.0022-202X.2005.23925.x 16297208

[B132] Zullo K. M. Guo Y. Cooke L. Jirau-Serrano X. Mangone M. Scotto L. (2014). The investigational aurora A kinase inhibitor alisertib exhibits broad activity in preclinical models of T-cell lymphoma and is highly synergistic with romidepsin. *Blood* 124:4493 10.1182/blood.V124.21.4493.4493

