# Supplementary material for: Recent Update of HDAC Inhibitors in Lymphoma
Source: Front Cell Dev Biol. 2020 Sep 3;8:576391. doi: 10.3389/fcell.2020.576391 (PMC7494784; doi:10.3389/fcell.2020.576391)
Supplement: Supplementary file 1 [file Table_1.DOCX]

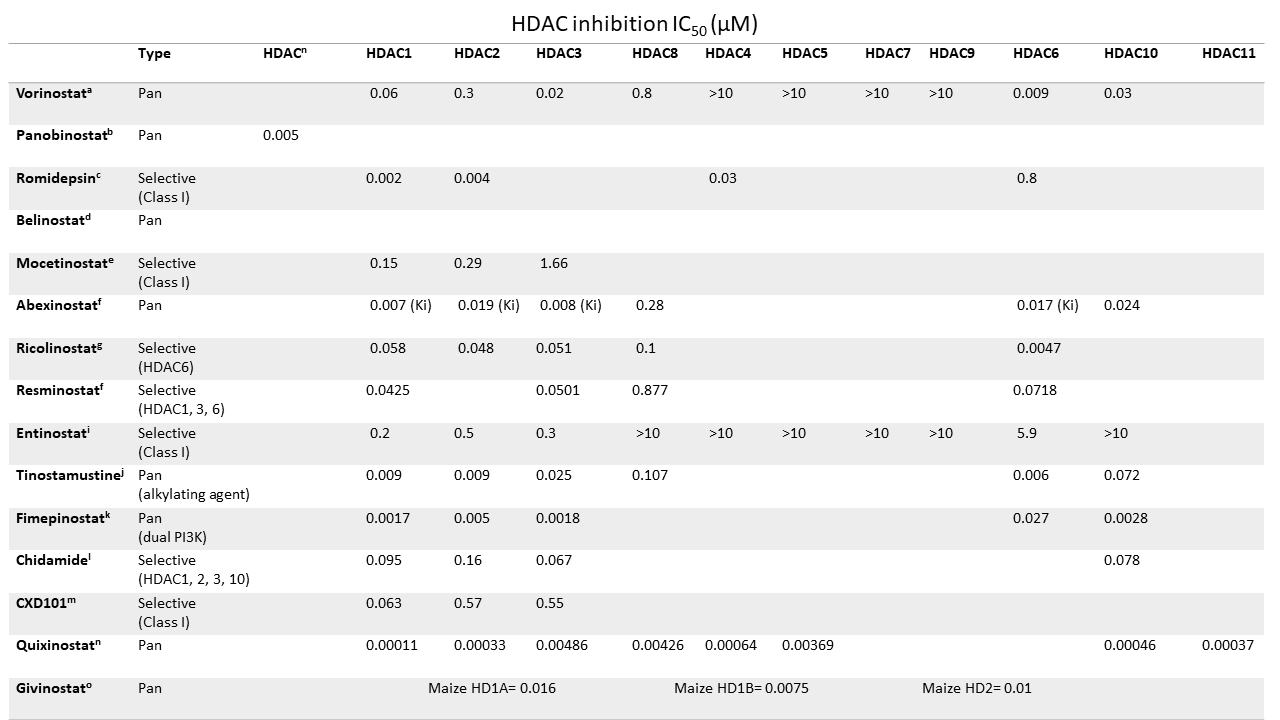


Table 1. List of HDAC inhibitor inhibition activity (IC_50_).

1. Wang, Y., Stowe, R. L., Pinello, C. E., Tian, G., Madoux, F., Li, D., et al. (2015). Identification of histone deacetylase inhibitors with benzoylhydrazide scaffold that selectively inhibit class I histone deacetylases. *Chem Biol*. 22, 273–284. doi: 10.1016/j.chembiol.2014.12.015
2. Scuto, A., Kirschbaum, M., Kowolik, C., Kretzner, L., Juhasz, A., Atadja, P., et al. (2008). The novel histone deacetylase inhibitor, LBH589, induces expression of DNA damage response genes and apoptosis in Ph- acute lymphoblastic leukemia cells. *Blood*. 111, 5093–5100. doi: 10.1182/blood-2007-10-117762
3. Newbold, A., Matthews, G.M., Bots, M., Cluse, L.A., Clarke, C.J., Banks, K.M., et al. (2013). Molecular and biologic analysis of histone deacetylase inhibitors with diverse specificities. *Mol. Cancer Ther.* 12, 2709–2721. doi: 10.1158/1535-7163.MCT-13-0626
4. Plumb, J. A., Finn, P. W., Williams, R. J., Bandara, M. J., Romero, M. R., Watkins, C. J., et al. (2003). Pharmacodynamic response and inhibition of growth of human tumor xenografts by the novel histone deacetylase inhibitor PXD101. *Mol Cancer Ther*. 2, 721–728.
5. Fournel, M., Bonfils, C., Hou, Y., Yan, P. T., Trachy-Bourget, M. C., Kalita, A., et al. (2008). MGCD0103, a novel isotype-selective histone deacetylase inhibitor, has broad spectrum antitumor activity in vitro and in vivo. *Mol Cancer Ther*. 7(4), 759–768. doi: 10.1158/1535-7163.MCT-07-2026
6. Buggy, J. J., Cao, Z. A., Bass, K. E., Verner, E., Balasubramanian, S., Liu, L., et al. (2006). CRA-024781: a novel synthetic inhibitor of histone deacetylase enzymes with antitumor activity in vitro and in vivo. *Mol Cancer Ther*. 5, 1309–1317. doi: 0.1158/1535-7163.MCT-05-0442
7. Santo, L., Hideshima, T., Kung, A. L., Tseng, J. C., Tamang, D., Yang, M., et al. (2012). Preclinical activity, pharmacodynamic, and pharmacokinetic properties of a selective HDAC6 inhibitor, ACY-1215, in combination with bortezomib in multiple myeloma. *Blood.* 119, 2579–2589. doi: 10.1182/blood-2011-10-387365
8. Mandl-Weber, S., Meinel, F. G., Jankowsky, R., Oduncu, F., Schmidmaier, R., and Baumann, P. (2010). The novel inhibitor of histone deacetylase resminostat (RAS2410) inhibits proliferation and induces apoptosis in multiple myeloma (MM) cells. *Br J Haemato.* 149, 518–528. doi: 10.1111/j.1365-2141.2010.08124.x
9. Lauffer, B.E., Mintzer, R., Fong, R., Mukund, S., Tam, C., Zilberleyb, I., et al. (2013). Histone deacetylase (HDAC) inhibitor kinetic rate constants correlate with cellular histone acetylation but not transcription and cell viability. *J. Biol. Chem*. 288, 26926–26943. doi: 10.1074/jbc.M113.490706.
10. Mehrling, T., and Chen, Y. (2016). The Alkylating-HDAC Inhibition Fusion Principle: Taking Chemotherapy to the Next Level with the First in Class Molecule EDO-S101. *Anticancer Agents Med Chem.* 16, 20–28. doi: 10.2174/1871520615666150518092027
11. Qian, C., Lai, C. J., Bao, R., Wang, D. G., Wang, J., Xu, G. X., et al. (2012). Cancer network disruption by a single molecule inhibitor targeting both histone deacetylase activity and phosphatidylinositol 3-kinase signaling. *Clin Cancer Res.* 18, 4104–4113. doi:10.1158/1078-0432.CCR-12-0055
12. Ning, Z. Q., Li, Z. B., Newman, M. J., Shan, S., Wang, X. H., Pan, D. S.,et al. (2012). Chidamide (CS055/HBI-8000): a new histone deacetylase inhibitor of the benzamide class with antitumor activity and the ability to enhance immune cell-mediated tumor cell cytotoxicity. *Cancer Chemother Pharmaco*. 69, 901–909. doi: 10.1007/s00280-011-1766-x
13. Eyre, T. A., Collins, G. P., Gupta, A., Coupe, N., Sheikh, S., Whittaker, J., et al. (2019). A phase 1 study to assess the safety, tolerability, and pharmacokinetics of CXD101 in patients with advanced cancer. *Cancer.* 125, 99–108. doi: 10.1002/cncr.31791
14. Arts, J., King, P., Mariën, A., Floren, W., Beliën, A., Janssen, L.,et al. (2009). JNJ-26481585, a novel "second-generation" oral histone deacetylase inhibitor, shows broad-spectrum preclinical antitumoral activity. *Clin Cancer Res*. 15, 6841–6851. doi: 10.1158/1078-0432.CCR-09-0547
15. Leoni, F., Fossati, G., Lewis, E. C., Lee, J. K., Porro, G., Pagani, P., et al. (2005). The histone deacetylase inhibitor ITF2357 reduces production of pro-inflammatory cytokines in vitro and systemic inflammation in vivo. *Mol Med.* 11, 1–15. doi: 10.2119/2006-00005.Dinarello
